# The Evolution of Mammalian Gene Families

**DOI:** 10.1371/journal.pone.0000085

**Published:** 2006-12-20

**Authors:** Jeffery P. Demuth, Tijl De Bie, Jason E. Stajich, Nello Cristianini, Matthew W. Hahn

**Affiliations:** 1 Department of Biology and School of Informatics, Indiana University Bloomington, Indiana, United States of America; 2 School of Electronics and Computer Science, ISIS Group, University of Southampton Southampton, United Kingdom; 3 Department of Molecular Genetics and Microbiology, Duke University Durham, North Carolina, United States of America; 4 Department of Statistics, University of California Davis Davis, California, United States of America; University of Chicago, United States of America

## Abstract

Gene families are groups of homologous genes that are likely to have highly similar functions. Differences in family size due to lineage-specific gene duplication and gene loss may provide clues to the evolutionary forces that have shaped mammalian genomes. Here we analyze the gene families contained within the whole genomes of human, chimpanzee, mouse, rat, and dog. In total we find that more than half of the 9,990 families present in the mammalian common ancestor have either expanded or contracted along at least one lineage. Additionally, we find that a large number of families are completely lost from one or more mammalian genomes, and a similar number of gene families have arisen subsequent to the mammalian common ancestor. Along the lineage leading to modern humans we infer the gain of 689 genes and the loss of 86 genes since the split from chimpanzees, including changes likely driven by adaptive natural selection. Our results imply that humans and chimpanzees differ by at least 6% (1,418 of 22,000 genes) in their complement of genes, which stands in stark contrast to the oft-cited 1.5% difference between orthologous nucleotide sequences. This genomic “revolving door” of gene gain and loss represents a large number of genetic differences separating humans from our closest relatives.

## Introduction

Explaining the obvious morphological, physiological, and behavioral traits that separate modern humans from our closest relatives, the chimpanzees, is challenging given the low level of nucleotide divergence between the two species [1]. More than 30 years have passed since King and Wilson first pointed out this apparent paradox, saying that “the genetic distance between humans and the chimpanzee is probably too small to account for their substantial organismal differences” [2]. To explain the paradox, King and Wilson proposed that regulatory changes rather than protein-coding mutations were responsible for the vast majority of observed biological differences [2]. Evidence gathered since that time demonstrates that amino acid [e.g. refs.1], [3], [4], [5] and regulatory sequence [6], [7] changes have both been involved in the evolution of uniquely human phenotypes.

A third source of differentiation, necessarily overlooked in comparison of orthologous sequences, is the differential duplication and deletion of chromosomal regions [8], [9]. Among human segmental duplications larger than 20 kilobases, 33% are not present in chimpanzee [10]. In total, it is estimated that at least 2.7% of the total genome has been uniquely duplicated subsequent to the human-chimpanzee split [10]; this number does not factor either deletions or small insertions into the total amount of divergence and therefore represents a minimum estimate. Per base pair, this translates into more than twice as many nucleotides unique to each species as there are nucleotide substitutions in orthologous sequences [11]. Without accounting for differences in the total DNA unique to each species, we cannot hope to take a proper accounting of the meaningful genetic divergence between humans and chimpanzees.

The most interesting duplication/deletion events from an evolutionary viewpoint are those that involve intact genes. Gene duplication has been hypothesized to be a powerful engine for evolutionary change in general [12], [13], and gene loss has been put forward as a common, advantageous response to changes in selective regimes in human history [14]. Recent gene duplicates are estimated to have arisen in the human genome at a rate of 0.009 /gene/million years (my) [15]. Using this rate, we would expect there to have been 1,188 new gene duplicates in the human genome since our split with chimpanzee (0.009 duplications/gene/my * 22,000 genes * 6 my). Assuming equal numbers of gene gains and losses and similar rates of turnover in chimps, the total number of genes in humans not present in chimps would be 2,376 (or ∼11% of all genes). This estimate of total genic divergence implied by rates of gene duplication has been widely overlooked due to the pervasive emphasis on nucleotide divergence between orthologous genes. Although this hypothesis assumes identical rates of gene gain and loss, and our coarse calculations have not considered that new gene duplicates are also the most likely genes to be lost, the consistency of gene number among fully sequenced mammals suggests that this is not an onerous assumption across short evolutionary time periods.

The process of differential gene gain and loss among species results in gene families that share sequence and functional homology but differ in gene number. Changes in gene family size have likely been important during human evolution [11], [16]–[20] and large differences in gene family size are generally ascribed to a selective advantage for either an increased or decreased gene number [19], [21]–[23]. While many of these differences may indeed be the result of natural selection, there has been little effort to account for the accumulation of differences due to random processes. For instance, a difference of 20 genes within a single family may be remarkable between human and chimpanzee, but not between human and mouse, or human and dog. Unlike the analysis of orthologous sequences, where there are widely accepted neutral expectations for molecular evolution [24], there has been no corresponding framework for the study of gene family evolution until recently [25].

The completed sequencing of multiple mammalian genomes provides unprecedented insight into the gain and loss of genetic material between species, and into the genomic changes exclusive to humans. In this paper we analyze gene gain and loss at a genomic scale by studying the expansion and contraction of gene families in the whole genomes of human, chimpanzee, mouse, rat, and dog. Using gene family assignments from the Ensembl project [26] (version 41 – October 2006; www.ensembl.org) we assign probabilities to the observed changes in gene family size along each mammalian lineage using a likelihood method that makes efficient use of genomic data in a phylogenetic context [25]. Our statistical framework provides a basis for improved inferences about causative evolutionary mechanisms by providing an expectation for the extent of variation in gene family size when gains and losses occur randomly. This means that we can identify branches of the phylogenetic tree where larger-than-expected contractions or expansions potentially indicate the action of adaptive natural selection [25].

Our investigation suggests that random processes explain most changes in gene family size; however, we find several families with larger than expected changes, including expansions in the human lineage for families with brain-specific functions. Additionally, we find that the total number of gene differences between humans and chimps estimated by our method is similar to that predicted above from independent analyses of recent segmental duplications. In total, our results support mounting evidence that gene duplication and loss may have played a greater role than nucleotide substitution in the evolution of uniquely human phenotypes, and certainly a greater role than has been widely appreciated.

## Results

### Rate of Change in Gene Family Size

**Figure 1:**
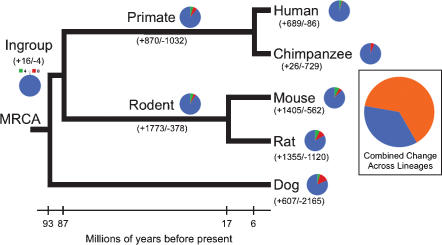
Distribution of gene gain and loss among mammalian lineages. Numbers in parentheses report number of genes gained or lost on each branch. Pie charts near branches show the proportion of families that expanded (green), contracted (red), or did not change (blue). The large pie chart shows the proportion of all families that change (orange), or remain constant (blue) across all lineages. Changes along long branches and on the ingroup branch may represent underestimates due to multiple gains and losses within individual families and or lack of phylogenetic resolution.

Over the 93 million years of mammalian evolution included in our analysis (Figure 1; Table 1), 56.3% (5,622/9,990) of gene families change size in at least one lineage. Despite this large amount of change, on average 90.2% of gene families do not change size along any particular branch of the phylogenetic tree (Table 2). The observed stasis of so many families along individual branches combined with the large proportion of families changing tree-wide indicates that most changes in gene family size are lineage-specific. Since the number of observed changes does not include families where equal numbers of gains and losses have occurred, our estimates represent minimum numbers of families that have changed in size during mammalian evolution.

**Table 1:**
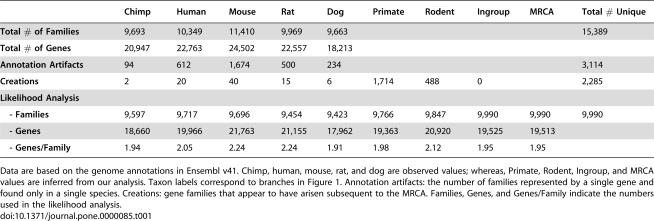
Numbers of genes and gene families in mammals.

	Chimp	Human	Mouse	Rat	Dog	Primate	Rodent	Ingroup	MRCA	Total # Unique
**Total # of Families**	9,693	10,349	11,410	9,969	9,663					15,389
**Total # of Genes**	20,947	22,763	24,502	22,557	18,213					
**Annotation Artifacts**	94	612	1,674	500	234					3,114
**Creations**	2	20	40	15	6	1,714	488	0		2,285
**Likelihood Analysis**										
**- Families**	9,597	9,717	9,696	9,454	9,423	9,766	9,847	9,990	9,990	9,990
**- Genes**	18,660	19,966	21,763	21,155	17,962	19,363	20,920	19,525	19,513	
**- Genes/Family**	1.94	2.05	2.24	2.24	1.91	1.98	2.12	1.95	1.95	

Data are based on the genome annotations in Ensembl v41. Chimp, human, mouse, rat, and dog are observed values; whereas, Primate, Rodent, Ingroup, and MRCA values are inferred from our analysis. Taxon labels correspond to branches in Figure 1. Annotation artifacts: the number of families represented by a single gene and found only in a single species. Creations: gene families that appear to have arisen subsequent to the MRCA. Families, Genes, and Genes/Family indicate the numbers used in the likelihood analysis.

**Table 2:**
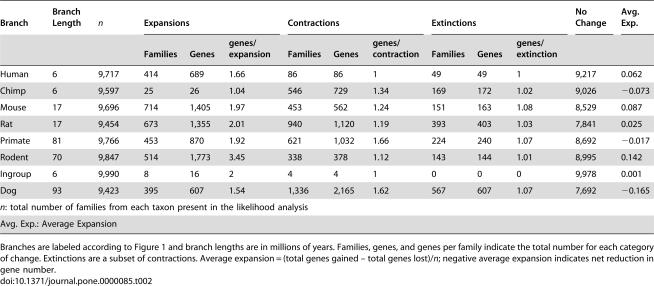
Changes in gene family size along each branch in the phylogenetic tree.

Branch	Branch Length	***n***	Expansions			Contractions			Extinctions			No Change	Avg. Exp.
			Families	Genes	genes/expansion	Families	Genes	genes/contraction	Families	Genes	genes/extinction		
Human	6	9,717	414	689	1.66	86	86	1	49	49	1	9,217	0.062
Chimp	6	9,597	25	26	1.04	546	729	1.34	169	172	1.02	9,026	−0.073
Mouse	17	9,696	714	1,405	1.97	453	562	1.24	151	163	1.08	8,529	0.087
Rat	17	9,454	673	1,355	2.01	940	1,120	1.19	393	403	1.03	7,841	0.025
Primate	81	9,766	453	870	1.92	621	1,032	1.66	224	240	1.07	8,692	−0.017
Rodent	70	9,847	514	1,773	3.45	338	378	1.12	143	144	1.01	8,995	0.142
Ingroup	6	9,990	8	16	2	4	4	1	0	0	0	9,978	0.001
Dog	93	9,423	395	607	1.54	1,336	2,165	1.62	567	607	1.07	7,692	−0.165
*n*: total number of families from each taxon present in the likelihood analysis													
Avg. Exp.: Average Expansion													

Branches are labeled according to Figure 1 and branch lengths are in millions of years. Families, genes, and genes per family indicate the total number for each category of change. Extinctions are a subset of contractions. Average expansion = (total genes gained – total genes lost)/*n*; negative average expansion indicates net reduction in gene number.

To account for unseen gains and losses, we estimated the average rate of change across all gene families via maximum likelihood. Based on the 9,990 families inferred to be present in the most recent common ancestor (MRCA) of mammals, our estimate for the average rate of genomic turnover is 0.0016 gains and losses per gene per million years. This number represents the probability that a given gene will give rise to a duplicate or be lost from the genome, and assumes that genomes are neither consistently expanding nor contracting. The assumption of no net gain or loss of genes is consistent with the observation that both total gene number and the distribution of family sizes remain relatively constant (Table 1) despite changes in the size of many individual gene families. Our estimate for the rate of gene gain and loss is similar to previously reported values for yeast [25] and is close to estimates for the birth rate of gene duplicates in mouse and rat (0.0013–0.0026 [27]), as well as several other non-human eukaryotes (0.001–0.016 [15]).

### Gain and Loss of Genes

While the likelihood estimate of the average rate of genome turnover assumes equal probability of gene gain and loss across the phylogenetic tree, for any given gene family or branch of the tree there may be more gains than losses (or vice versa). To map changes in gene family size onto individual branches of the tree, and to determine whether changes are expansions or contractions [cf. refs.23], [28], we obtained maximum likelihood estimates of ancestral gene family sizes across the mammalian tree. Changes on each branch were then calculated by taking the difference in the size of families between parent and daughter nodes.

In total, expansions outnumber contractions on the human, chimp, mouse, rodent, and ingroup branches (Table 2). The mouse lineage has the largest number of gene family expansions (714 families), while the rodent lineage has the largest gain in gene number via expansion (1,773 genes) [29]. Contractions predominate on the primate, rat, and dog branches. Dog has the smallest number of genes in its genome, as well as the largest number of gene family contractions (1,336) and losses of genes (2,165). Thus the equal probability of gain and loss in our likelihood model has clearly not constrained our inference of overall genome size change in this species. Along the lineage leading to humans, 414 families have expanded and 86 have contracted (Table 2). These changes account for the gain of 689 genes and the loss of 86 genes from the human genome. Over the same time period, chimpanzees have experienced expansions in 25 families (26 genes) and contractions in 546 families (729 genes).

In order to investigate the impacts of ongoing refinements to genome annotations and the similarity threshold used for defining gene families, we conducted two extensive checks on our results. To investigate the effects of revised annotation, we conducted many of the same analyses carried out on Ensembl v41 on previous versions 32, 35, 38, and 39. In addition to numerous minor gene annotation updates to all of the genomes, these five Ensembl versions represent major assembly or annotation changes for human, mouse, and chimpanzee. Analyses of versions prior to v41 yielded highly similar results: the numbers of gains and losses remain similar across annotations, as do the overall number of genes that differ among species (Table S1). These versions include major changes to the human and mouse genome annotations. The major revision of the chimpanzee genome in v41 represents a substantial improvement in sequencing coverage for that species from ∼4× to ∼6×. As a result, the difference in gene complement between humans and chimpanzees decreases by ∼1.5 fold (∼2,382 in v32-39 to 1,418 in v41). Our estimate for the average rate of gene gain and loss across mammals is impacted less by the improved chimpanzee assembly, changing from 0.0022 in v32-39 to 0.0016 in v41.

To assess the impact of different clustering thresholds used to define gene families on our inferences of gene gain and loss, we obtained the raw protein distance data used to construct gene families from Ensembl (A. Ureta-Vidal, pers. comm.). Using an arbitrary subset of the data (∼90,000 genes) —for computational tractability—we mimicked the Ensembl pipeline to generate our own gene families using the program MCL [30]. We ran MCL with five different values of the clustering parameter, including the value used by Ensembl, to generate families with average amino acid similarities both higher and lower than the original data. Across these new gene family datasets, the number of families and their size changed predictably with the clustering threshold: higher similarity cut-offs resulted in a greater number of individually smaller families, while lower cut-offs resulted in fewer, larger families. Most importantly, however, the estimated differences in gene complement between human and chimp changed by a maximum of only 0.41% from the value found using the original Ensembl clustering parameter (Table S1). Thus, even though individual families include more or fewer genes, the patterns of change are highly consistent across parameter values such that our conclusions remain qualitatively unaffected.

### Loss of Gene Families

Summing across all of the inferred family sizes at the root of the tree, we estimate that the genome of the mammalian MRCA contained at least 19,513 genes. This estimate may be low given that more genes may have been present in gene families that no longer exist in extant taxa. Indeed, many families that experience contractions are lost completely on one or more branches of the phylogenetic tree [e.g. ref. 31]. These “extinctions” occur on almost every branch of the tree (Table 2), and include genes involved in a wide variety of biological functions. In total there are 1,421 families inferred to have been present in the mammalian MRCA that have zero genes in at least one extant genome.

The most common functional categories of extinct families involve immune response, chemosensation, reproduction, and transcription (but note that the function of 36.4% were categorized as ambiguous or unknown). A complete list of extinct families is available as Table S2 online. We found 289 families present in the mammalian MRCA that appear to have been lost from the human genome. Of these losses, 240 are shared with chimpanzees and 49 are unique to the human lineage (Table 2). The human lineage has the fewest number, but greatest proportion of extinctions, with 49 (48.3%) of 86 contractions resulting in gene family extinction. The dog genome has lost the largest number of families since the mammalian MRCA, with 567.

For each extinction the average number of genes lost on the terminal lineage is approximately 1.0 (Table 2). While this value does not indicate the total distance traversed to extinction, it does show that most extinctions do not involve the sudden loss of many genes. Among extinct gene families in humans, the largest families in the mammalian MRCA are both inferred to have contained only two genes. However, the largest family in the mammalian MRCA to eventually go extinct in any lineage (11 genes) is a V2R vomeronasal receptor gene family that has >100 genes in each rodent lineage but is absent from the dog, human, and chimpanzee genomes (it is, however, present in the preliminary assembly of the rhesus macaque genome; see Materials and Methods).

The apparent loss of whole gene families can result from several factors, including: 1) the true loss of all genes from a genome due to deletion or pseudogenization; 2) the rapid evolution of protein sequences, such that the genes are no longer identified as belonging to the same family; or 3) losses may be an artifact of the threshold used for clustering. The types of families that are lost most commonly in our analysis have also been shown to have elevated rates of nonsynonymous substitution [32], which suggests that some families that we have counted as “extinct” may simply be highly diverged in a single lineage.

Inferences of complete gene family loss depend on the similarity threshold used to define families; if a lower threshold is used, then families are larger and proportionally fewer of them are expected to be lost [31]. Using the re-clustered Ensembl gene families described in the previous section, we find that the clustering parameter has a significant effect on the number of gene family extinctions (r^2^ = 0.976, *P* = 0.004). However, even in the situation where families are individually quite large, there are still extinctions of entire gene families (Figure S1).

### Lineage-specific Gene Families

In addition to the families inferred to be present in the 93 million year-old MRCA, 2,278 gene families are found in only a subset of extant taxa and likely arose more recently (Table 1; “Creations”). Because our likelihood model assumes that each family is present in the MRCA, we analyzed the gene families with more recent origins separately from those discussed above. The largest fraction of these (1,730/2,278 families) are found only in both human and chimpanzee (Table 1). There are an additional 20 families containing greater than one gene found only in the human genome and 2 found only in the chimpanzee genome. The much larger number of lineage-specific families in humans and primates as a whole may be due to the fact that much of the genome annotations of the other mammalian species were transferred directly from the human genome [1]. The complete list of creations, including gene distributions among taxa, is available as Table S3 online.

The largest family created along the primate lineage has 46 chimpanzee and 63 human genes. One of the few genes in this family where functional data are available is caspase-7. Genes in the caspase-dependent apoptosis pathway, of which caspase-7 is a part, are critical in mammalian brain and neuronal development. Furthermore, genes in this pathway have undergone accelerated protein sequence evolution in primates—especially in humans —that is consistent with positive natural selection [33].

Creations in rodents are clustered into fewer but larger families than in primates (1,963 genes in 542 families). In dog there are only six, relatively small, unique families. The largest of these families, submaxillary mucin, contains five genes total and a single characterized salivary protein. Across all taxa, the proportion of families with unknown or ambiguous function is twice as large for creations (1,192 unknown+360 ambiguous/2,278 total creations = 68.1%) as for families included in the full statistical analysis (896 unknown+1,586 ambiguous/9,990 total families = 24.8%).

There are multiple biological processes that may be responsible for the creation of new gene families [23]. These include: the creation of new genes (through both gene fusion and *de novo* origination [34]), accelerated nucleotide divergence of a new duplicate or member of a previously existing gene family [35], and/or horizontal gene transfer [36]. While many newly arisen gene families have unknown or ambiguous function, many of them can be functionally annotated via similarity to pre-existing families — though the low level of similarity between them may preclude placing them in the same family. Additionally, even families where the consensus function is “unknown” often contain at least one gene with known function, as in the case of the family containing the primate caspase-7 gene. These pieces of evidence suggest that creations are largely due, at least in large part, to divergence of young duplicates from previously existing families.

Creations may also arise as an artifact of the gene family clustering. If creations were solely an artifact of the clustering threshold, we would expect there to be a correlation between the number of creations in each lineage and the number of contractions (including extinctions) in that lineage. The number of creations and contractions within lineages are not significantly correlated (*r* = −0.23, *P* = 0.59). To further investigate the effect of clustering threshold on our inference of gene family creations, we analyzed the re-clustered Ensembl gene families as we did above for extinctions. As with extinctions, the clustering parameter has a significant effect on the number of gene family creations (r^2^ = 0.983, *P* = 0.003). Not surprisingly, the absolute number of inferred family creations depends on the similarity cut-off used: if a lower threshold is used then the absolute number of families decreases, and consequently there are proportionally fewer creations of entire families. Note, again that even in the situation where families are quite large there are still many creations of entire gene families (Figure S1).

### Accelerated Evolution of Gene Families

**Figure 2:**
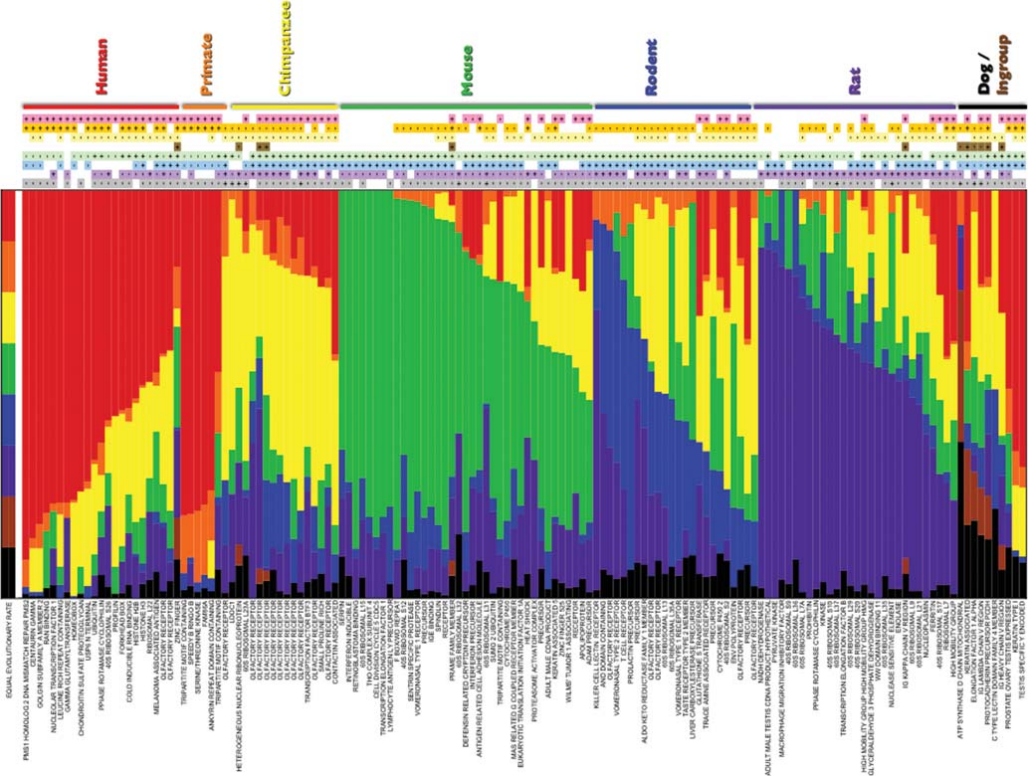
Rapidly evolving gene families in mammals. Horizontal bars indicate the relative rates of change among taxa for each family (where rate is the change in number of genes per million years). The top bar indicates the hypothetical case where each lineage has an equal evolutionary rate. Bars are partitioned by the color codes assigned to the taxon names at the far right. Boxes immediately right of each bar indicate whether changes in that family are expansions (+) or contractions (−) in each taxon. Each column of boxes represents a single taxon, color coded in the same order as the bars. Vertical bars on the right span families that were found to be least likely in the same lineage under the random model of gene gain and loss. Families with ambiguous or unknown function are left out of the figure to improve legibility. The complete statistical results are presented in Table S7.

Considering only the 9,990 gene families present in the MRCA of mammals, we found 164 to be evolving non-randomly at *P* <0.0001 (Figure 2). At this cut-off we expect zero families to be significant by chance, resulting in a false discovery rate [37] of 0.01% for this set of rapidly-evolving gene families. The most common biological functions assigned to these gene families include immune defense and response, transcription, translation, brain and neuron development, intercellular communication and transport, reproduction, and metabolism. Interestingly, comparisons of both nonsynonymous to synonymous nucleotide divergence and regulatory sequence divergence have also shown that genes with these biological functions are evolving rapidly in mammals [1], [6], [27], [38], [39]. This implies that natural selection may act at a multiplicity of levels during adaptive molecular evolution.

For each of the significant gene families, we identified the branch of the phylogenetic tree that showed the most unlikely acceleration in genomic turnover (Figure 2; see Materials and Methods). We find 30 families where the lineage leading to modern humans contains the most significant departure from random gain and loss of genes. One of the families evolving significantly faster than expected in humans contains the gene *centaurin gamma 2*, which has been implicated in the genetic etiology of autism [40]. This family has 15 genes in humans and no more than 6 genes in any of the other extant mammals. The large expansion along the human lineage implies the gain of at least 8 genes in the last six million years.

Other gene families that are evolving rapidly along the human lineage include significant expansions in a forkhead box transcription factor family and a golgin subfamily. Language acquisition in humans has been tied to a rapidly-evolving forkhead box protein, *foxp2* [3], although this particular gene is in a different forkhead family. The golgins are a group of coiled-coil proteins responsible for tethering specific molecules for transport through the golgi complex [41], and have been implicated in systemic autoimmune diseases such as lupus erythematosus and Sjögren's syndrome [42]. This particular golgin subfamily has 49 human genes, 23 chimpanzee genes, and only a single copy each in the rodents and dog.

One concern may be that our likelihood approach does not accurately estimate the number of gains and losses when there are large changes in gene family size. To investigate the extent that our method is accurately estimating the number of changes, we generated gene trees for the three rapidly-evolving families discussed in the preceding paragraphs (see Materials and Methods and Figures S2–S4). Reconciling these gene trees with the 5-taxon species tree (Figure 1) provides an alternative method for estimating gene gain and loss [43]. Overall, there is a strong agreement between the results produced by the likelihood and gene tree methods: the correlations between gains and losses for all branches of the tree are *r* = 1.0 (*P*<0.00001) for centaurin gamma, *r* = 0.85 (*P* = 0.007) for forkhead box, and *r* = 0.80 (*P* = 0.016) for the golgins (there are multiple lineages with zero changes using both methods in this last family, resulting in a slightly lower *P*-value). Along the lineage leading to humans—where the largest changes occur—both methods agree exactly for the centaurin gamma and forkhead box families, while the likelihood method underestimates the number of gene gains relative to the gene tree method for the golgin family. This underestimation is expected when either multiple gains and losses occur on an individual branch, or parallel gains or losses occur on multiple branches [25]. These results indicate that the likelihood method used here is highly accurate, though it may sometimes give an underestimate of the number of changes along individual lineages.

Interestingly, all of the most significant human families are expansions (Figure 2). If large expansions in family size are more likely the result of natural selection than are large contractions [23], [44], then our results suggest that many of the significant changes in human are due to adaptive evolution. While this does not mean that any single gene gain or loss has not been driven by natural selection, it does indicate that gene gain may be a likely instrument of adaptation. It should be noted, however, that large expansions in the size of gene families could also be due to either increased rates of gene duplication via mutation in individual families, or some form of relaxed selection that allows for increased rates of fixation of duplicate genes. We favor an interpretation of positive selection, though definitive evidence will have to await further studies.

In contrast to the potential for gene family expansion via natural selection, there is a well-documented loss of olfactory receptors (ORs) in primates that may be due to relaxed selection on odorant perception coincident with the acquisition of trichromatic vision [45]; relaxed selection may also be responsible for large losses in other families. Significant contractions of OR gene families are also found by our analyses, and thus act as a positive control for the statistical methodology used here. Olfactory receptors are the largest gene superfamily in mammals, with each subfamily being responsible for detecting hundreds of odor molecules [17], [46]. In humans and chimpanzees it is known that most ORs are present in the genome as non-functional pseudogenes [47]. This is reflected in the distribution of genes in the extant genomes, where the rodent species have more than twice as many OR genes as the primate species: dog (539), rat (1,192), mouse (1,128), chimpanzee (335), human (494). In our analysis 18 of 26 OR sub-families contain significant changes in size (Figure 2). For each of the families, highly unlikely changes occur on more than one branch. The MRCA of mammals is inferred to have had 660 ORs total, and the predominant pattern has been one of expansions in rodents and contractions in primates. This agrees with recent results showing that differences between humans and mice are a result of both accelerated loss in humans and addition of functional OR genes in mice [48].

We also observed the co-evolution of several functionally related gene families. The proteins that make up the 40S and 60S ribosomal subunits show a coordinated expansion along the lineages leading to mouse and rat. Of the 20 significant expansions involving ribosome-related gene families, 18 represent changes on the rodent, mouse, or rat lineages (Figure 2). Ribosomes are the site of protein synthesis in all organisms; increases in ribosome-associated proteins may therefore be the result of selection for increased reproductive rate and/or shorter generation time [49]. An alternative to this adaptive hypothesis is the possibility that a high rate of ribosomal protein retroposition has left many intact, but non-functional copies in the two rodent genomes, though the rate of retroposition for this class of genes is in fact increased in humans relative to mouse [50].

To ensure the robustness of our inferences of accelerated gene family evolution, we again identified families with *P*<0.0001 in Ensembl v39 and v32. To the extent that homologous families can be identified, results among the different annotations are highly congruent (Table S4). Thus, our re-analysis of rapidly evolving families indicates that our approach remains robust to the level of revision in these annotations, which includes major updates of human, mouse, and chimpanzee genome assemblies.

## Discussion

Our analyses demonstrate a high rate of genomic turnover at the level of gene gain and loss. This genomic “revolving door” of genes entering genomes just as genes are leaving has important implications for the number of differences between closely related species. Our results indicate that the human genome contains 1,418 genes—6.4% of all genes—that do not have orthologs in the chimpanzee genome (689 gains in humans+729 losses in chimpanzee/22,000 total genes). This difference is similar to the proportion of large duplicated regions that are unique to each species (2.7%) [10], as well as to estimates of divergence that consider both smaller insertions and deletions (5%) [8]. Furthermore, if we include differences in the size of gene families that are unique to the primates (such that we cannot polarize changes as gains or losses), this would add an additional 566 genes that do not have orthologs between the two species (Table S3). Similar calculations between rat and mouse reveal that approximately 10% of genes between these two species are not orthologs (Table 2). These estimates are consistent with elevated rates of duplication in hominoids and Old World monkeys relative to New World monkeys and other fully sequenced animals [11], [18].

Recent studies of gene copy-number polymorphism also provide evidence for the presence of a large number of segregating gene duplications and deletions in humans [51], [52]; many of these genes are even in the same families that we find to be evolving at the highest rates in humans [52], [53]. Our results therefore support a growing appreciation for the importance of duplication and deletion events in the course of human evolution [9], [14].

There are two primary sources of potential bias in our study. First, differential sequencing coverage and genome assembly quality may inflate our estimates of gene gain and loss. To minimize this source of bias we focused only on mammalian genomes that were estimated to be >90% complete (rat >90% [27], chimpanzee >94% [1], mouse ≈96% [38], dog ≈99% [54], human ≈99% [39]). Our analyses of multiple versions of genome annotations showed that the most dramatic change in perceived gene gain and loss occurred as a result of the 1.5× increase in chimpanzee genome sequencing coverage. The pattern of gene gain and loss between humans and chimpanzees is in the direction expected if many genes remain unidentified in the PanTro2.1 genome assembly (i.e. more gains in humans and losses in chimps).

The total difference in gene number between these two species cannot explain all of the divergence in gene complement that we observe. Even if all of the unidentified genes in chimpanzee contributed to reducing the number of differences between these two species (such that there were equal numbers of genes in the two genomes), there would still be a 2% difference in the gene complement between humans and chimps (∼400 genes). Ultimately judging the extent to which our results are impacted by heterogeneous data quality will require comparison with future refinements of the genome sequences and annotations. Our analyses do suggest that comprehensive identification of the genetic changes that underlie species differences will require genome sequencing deeper than 6× coverage in order to fully account for duplication and loss events. In the unlikely event that revised annotations significantly reduce the estimated difference between human and chimpanzee presented here, our results would still suggest an important and widely unappreciated contribution of gene gain and loss to human evolution.

A second concern is that the level of similarity used to define gene families affects the size of families, and stricter criteria result in more apparent extinctions and creations of entire families [31]. Conversely, allowing gene families to include highly-diverged members will necessarily result in larger families and fewer extinctions and creations. While there is no one accepted similarity criterion for the definition of gene families, differences in the relative numbers of creations and extinctions among lineages (as well as single gene gains and losses) should not be affected by the definition used. Indeed, the number of creations and extinctions are highly correlated across similarity thresholds (*r* = 0.995, *P* = 0.0004). Our results provide evidence for a high number of extinctions and creations of whole gene families, no matter how families are defined. We expect that commensurately many of both types of events will be found in future analyses, regardless of the exact definitions of gene families.

Because our analyses attempt to give a genome-wide perspective on gene family evolution, the precision of genic divergence estimates will necessarily reflect the accuracy of the underlying genome sequences and annotations. In the interest of transparency, we use widely-accepted and freely-accessible genome annotations as the foundation for our analyses [26]. In most cases sequencing and annotation errors are likely to inflate estimates of change; however, the simple method we have used to count differences in gene number is fundamentally conservative because we are unable to account for instances where both gains and losses occur within a family along the same branch of the tree. Careful individual analysis of each gene family is sure to increase the number of both gains and losses on every branch of the tree.

Although alignment of orthologous genomic regions has shown that there is less than 2% divergence between humans and chimpanzees at the nucleotide level [1], these analyses perforce do not consider unalignable or ambiguously-aligned regions [8]. In contrast, our results demonstrate that humans and chimpanzees differ by ∼6% at the level of gene complement. Though recent work has focused on distinguishing amino acid changes from regulatory changes as the major determinants of human evolution [55], we have shown here that the similarity in total gene number between humans and our closest relatives has masked the gain and loss of genes as a fertile source of adaptive change.

## Materials and Methods

### Data collection

Gene families were assembled by the Ensembl project [26] (version 41 – October 2006; www.ensembl.org) using the MCL algorithm. Briefly, MCL uses a Markov clustering algorithm to cluster proteins into families by simultaneous analysis of sequence similarities among all genes in all taxa. The approach overcomes many of the difficulties that arise when attempting pair-wise clustering of proteins with complex domain structures [30]. Furthermore, because clustering proceeds irrespective of the species-of-origin, the resulting assignments represent an objective measure of the number of genes in each family, for each species. We assembled a matrix of gene family sizes for: *Canis familiaris* (dog), *Rattus norvegicus* (rat), *Mus musculus* (mouse), *Pan troglodytes* (chimp), and *Homo sapiens* (human), for analysis of mammalian gene family evolution. We included only the longest isoform of each gene in our analysis.

The resulting matrix includes 15,389 gene families. Among these families, 3,114 (20.3%) are species-specific, single-gene families. We consider these to be artifacts of gene prediction and to minimize this source of bias, we exclude them from our analyses. The remaining dataset consisted of 12,225 gene families present in at least two species, or composed of greater than one gene in a single species (Table 1). The mouse genome, which initially appeared to have the largest number of genes and gene families, contains the highest proportion of these “annotation artifacts” (1,674 of 11,370 mouse families). The list of families deemed to be artifacts is available as Table S5 online.

### Likelihood analysis of gene gain and loss

To identify gene families that have undergone significant expansion or contraction and to estimate the global rates of gene gain and loss, we applied the probabilistic framework developed by Hahn et al. [25]. The method models gene family evolution as a stochastic birth and death process, taking into account the phylogenetic tree topology and branch lengths. Assuming that all genes have equal probability, *λ*, of gain (birth) or loss (death), the conditional probability of going from an initial number of genes, *X_0_ = s*, to size *c* during time *t*, *X_t_ = c*, is given by
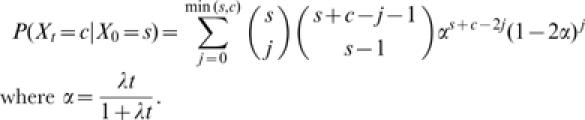
.

Note that if *X_0_* = 0 then there is no chance of birth or death, as 0 is an absorbing boundary; therefore we restrict our analysis to *X_0_*>0. The assumption of the stochastic birth-death model that each family is present in the MRCA (i.e. *X_0_*>0), means that we excluded newly created families from our estimation of *λ*.

For gene families inferred to be present in the MRCA of mammals, family sizes at the internal nodes are computed while maximizing the likelihood of the observed family sizes at the tips. This maximization involves averaging over all possible values of unspecified internal nodes except for the root node, *X_0_*, on which each iteration is conditioned. Software used to conduct the analysis performed above is publicly available (www.bio.indiana.edu/∼hahnlab/Software.html; [56]).

Details specific to the analysis of mammalian genomes are as follows: The topology and branch lengths of the phylogenetic tree used are taken from the average values reported by Springer et al. [57] based on 16,397 aligned nucleotides of 19 nuclear and three mitochondrial genes. We then estimated *λ* using all 9,990 families present in the mammal MRCA (Table S6). Branch identification for significant families was not computed on the full data set due to computing time constraints. To be conservative we first determined families significant at a false discovery rate [37] of 0.01% (i.e. *P*<0.0001). For the 164 families with tree-wide *P*<0.0001, we then specified use of the estimated *λ* while calculating branch-cutting, Viterbi, and likelihood ratio tests [25]. This final step required approximately 6.5 days to compute on a dual processor 2.7 GHz PowerMac G5. In the end, all three methods yielded congruent results (see Table S7 for individual results from all three methods).

### Distinguishing creations and extinctions

Based on the distribution of family presence in the leaf taxa, we assign creations to specific branches of the tree using a parsimony rule where: if two branches share a family, a creation is assigned to the MRCA of those branches. To illustrate, a family shared by only humans and chimpanzees is assigned a creation on the primate branch. In order to ensure that creations were not simply extinctions in a majority of the species studied here, we further queried the other eukaryotic genomes present in Ensembl v.41. These genomes included *Saccharomyces cerevisiae, Ciona savignyi, Caenorhabditis elegans, Anopheles gambiae, Ciona intestinalis, Drosophila melanogaster, Danio rerio, Xenopus tropicalis, Gallus gallus, Macaca mulatta, Bos taurus, Monodelphis domestica, Takifugu rubripes,* and *Tetraodon nigroviridis*. In total, 611 gene families were found in further outgroup taxa and represent a minimum number of additional extinctions that occurred in the majority of lineages included in our full analysis. Excluding families found in one of the 14 more distantly-related eukaryotes as well as transposable elements and retroviruses leaves 1,317 gene families that appear within the past 93 million years. To investigate the correlation between gene family creations and gene family contractions (see *Lineage-specific Gene Families* above), we considered the total number of each type of event on every branch of the tree separately (data from Table 2).

### Effects of annotation changes and clustering criterion

To assess the effects of genome annotation updates, many of the analyses conducted on Ensembl v41 (October 2006) were also conducted on the following versions of Ensembl: v32 (July 2005), v35 (November 2005), v38 (April 2006), and v39 (June 2006). Results for changes in gene family size are available in Table S1. To assess the effects of clustering threshold, we used MCL to cluster a subset of the data obtained directly from Ensembl containing 89,562 genes (A. Ureta-Vidal, pers. comm.). The clustering parameter, *i*, values used were: 1.5, 1.9, 2.3, 2.7, and 3.1. The Ensembl database uses *i* = 2.3 to define gene families. Clustering threshold has a strong influence on the absolute number of families, as well as on the number of creations and extinctions (Figure S1). In order to see whether the clustering parameter affects the number of differences inferred between human and chimpanzee, we calculated the number of differences between these two taxa at *i* = 2.3. We then compared this value to the number of differences estimated from the four other clustering parameter values used (Table S1).

### Reconciling gene trees and species trees

For the forkhead box (ENSF00000000311), centaurin gamma (ENSF00000000936), and golgin (ENSF00000000597) families, we downloaded protein sequences for all genes in all five mammalian species. We then generated neighbor-joining trees in PHYLIP using JTT protein distances [58]. We reconciled the resulting gene tree with the 5-taxon species tree described above using the NOTUNG software package [59].

## Supporting Information

Figure S1 The effect of clustering threshold on total number of families and the correlation with creations and extinctions. r2 is reported for the correlation between total number of families and the number of creations or extinctions. i values represent the clustering threshold (in MCL) responsible for the corresponding numbers of gene families, creations, and extinctions.
(0.08 MB TIF)

Figure S2 Gene tree for the forkhead box gene family (ENSF00000000311), showing gene duplication events as red boxes (H = human, C = chimp, M = mouse, R = rat, D = dog).
(6.70 MB TIF)

Figure S3 Gene tree for the centaurin gamma gene family (ENSF00000000936), showing gene duplication events as red boxes (H = human, C = chimp, M = mouse, R = rat, D = dog).
(8.27 MB TIF)

Figure S4 Gene tree for the golgin gene family (ENSF00000000597), showing gene duplication events as red boxes (H = human, C = chimp, M = mouse, R = rat, D = dog).
(4.09 MB TIF)

Table S1 Supporting analyses of the effects of gene family clustering thresholds and genome annotation updates.
(0.02 MB XLS)

Table S2 Families that have gone extinct in one or more lineages.
(0.24 MB XLS)

Table S3 Families inferred to have arisen after the MRCA of the taxa included in our analyses. The first column describes whether the family on each row was found in the 14 other eukaryotes we queried to verify our inferences. Ingroup creations are those that are present in 3 or 4 of the mouse, rat, human, chimpanzee group, but absent in dog and the other 14 eukaryotes.
(0.34 MB XLS)

Table S4 Comparison of rapidly evolving families found in Ensembl versions 32, 39, and 41.
(0.03 MB XLS)

Table S5 Species-specific single-gene families. We considered these to be annotation artifacts and excluded them from analyses.
(0.48 MB XLS)

Table S6 The full list of families included in the likelihood analysis of gene family evolution. The table includes the inferred gene family sizes at the ancestral nodes.
(1.75 MB XLS)

Table S7 The 164 families that show individually accelerated rates of evolution. The table contains results from all three methods of identifying the branch that is least likely to be evolving randomly.
(0.14 MB XLS)
