# Supplementary figures and images for: The Evolution of Mammalian Gene Families
Source: PLoS One. 2006 Dec 20;1(1):e85. doi: 10.1371/journal.pone.0000085 (PMC1762380; doi:10.1371/journal.pone.0000085)

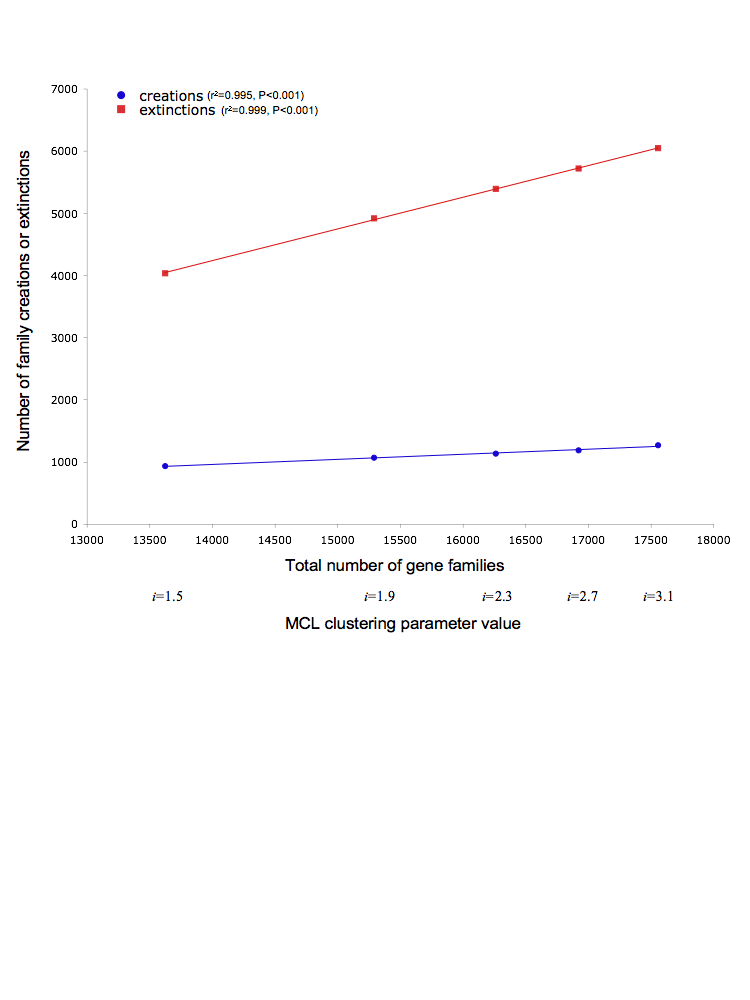

Supplement: Figure S1 — The effect of clustering threshold on total number of families and the correlation with creations and extinctions. r2 is reported for the correlation between total number of families and the number of creations or extinctions. i values represent the clustering threshold (in MCL) responsible for the corresponding numbers of gene families, creations, and extinctions. (0.08 MB TIF) [file pone.0000085.s001.tif]

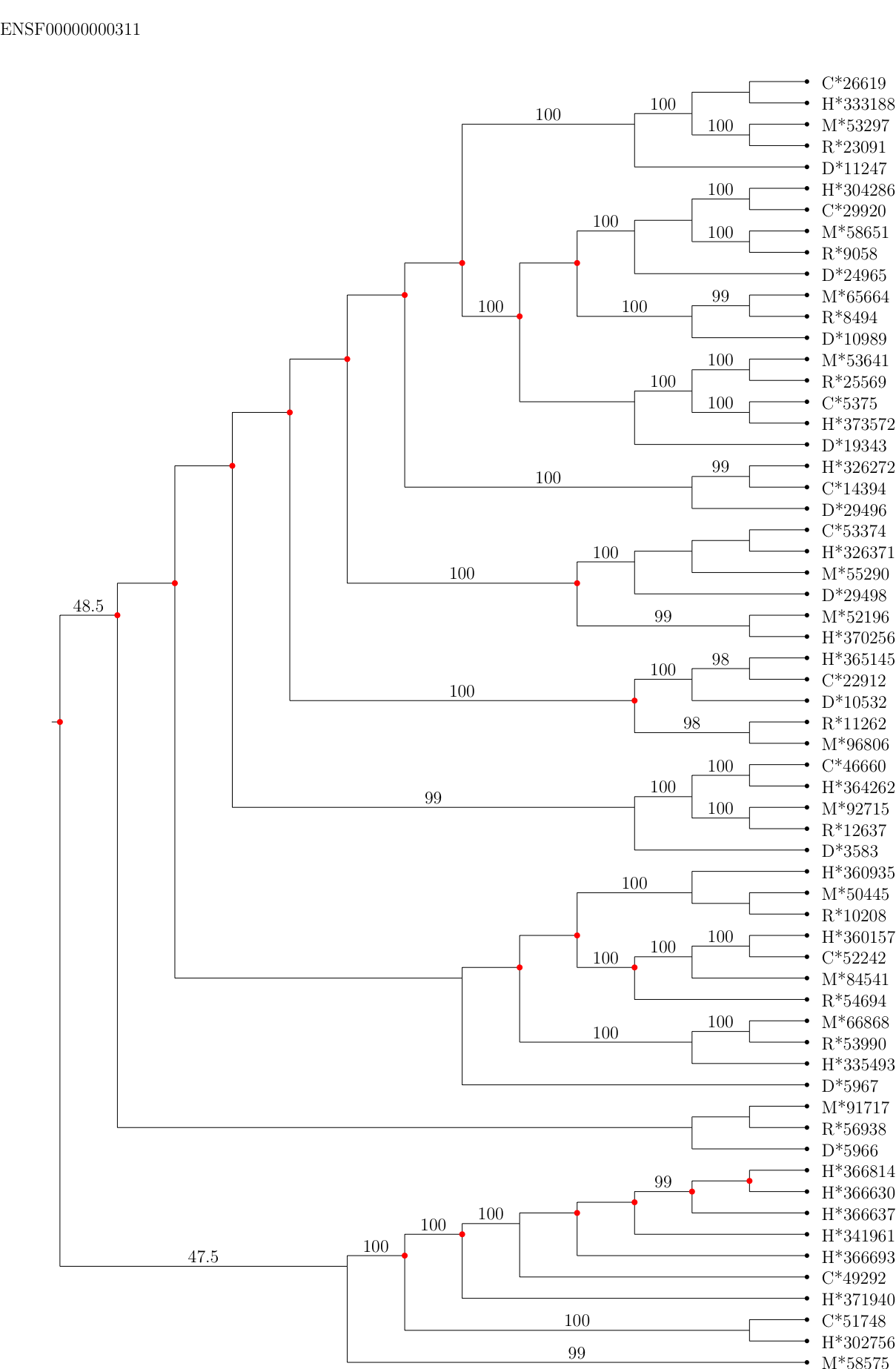

Supplement: Figure S2 — Gene tree for the forkhead box gene family (ENSF00000000311), showing gene duplication events as red boxes (H = human, C = chimp, M = mouse, R = rat, D = dog). (6.70 MB TIF) [file pone.0000085.s002.tif]

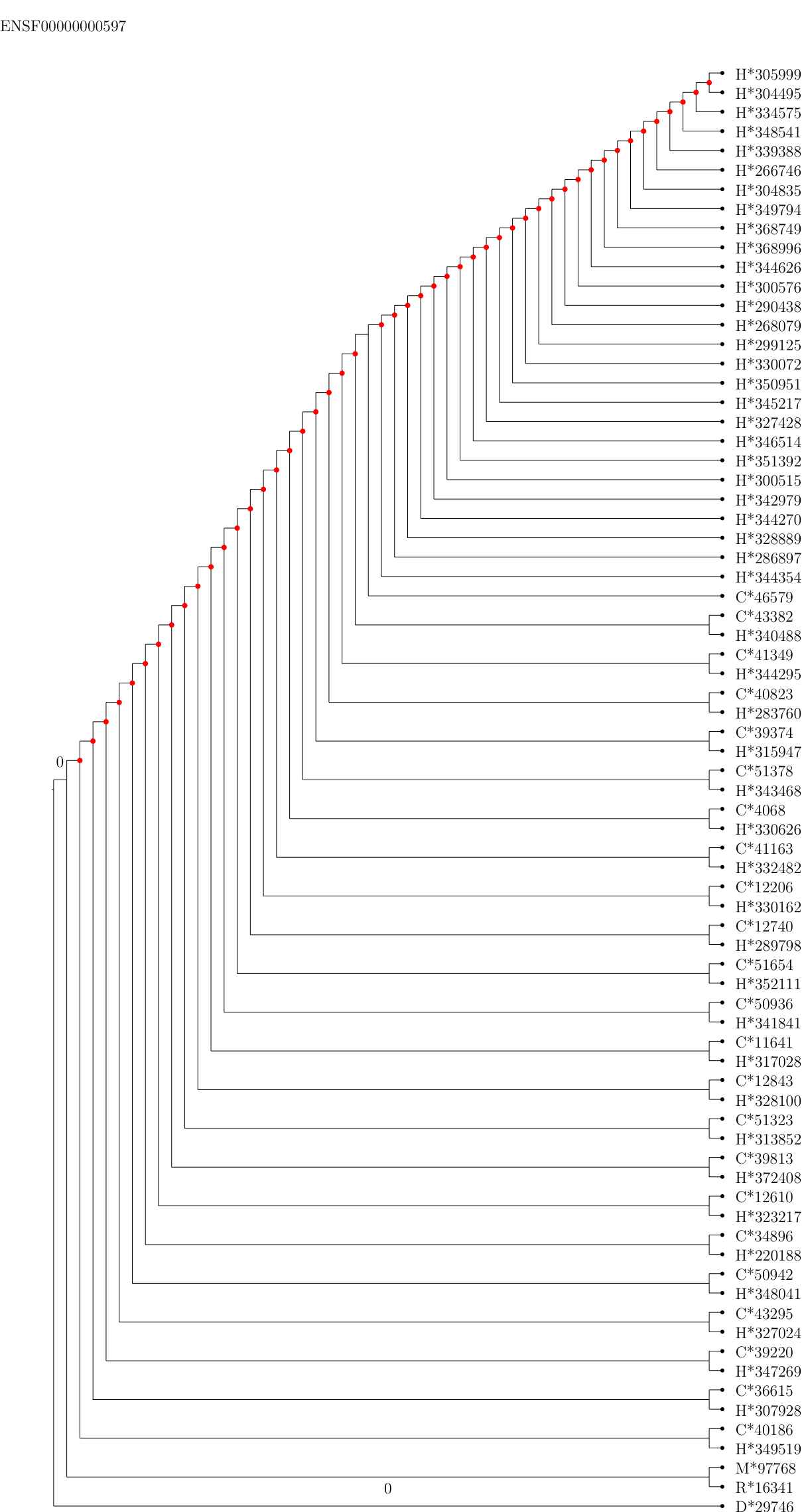

Supplement: Figure S3 — Gene tree for the centaurin gamma gene family (ENSF00000000936), showing gene duplication events as red boxes (H = human, C = chimp, M = mouse, R = rat, D = dog). (8.27 MB TIF) [file pone.0000085.s003.tif]

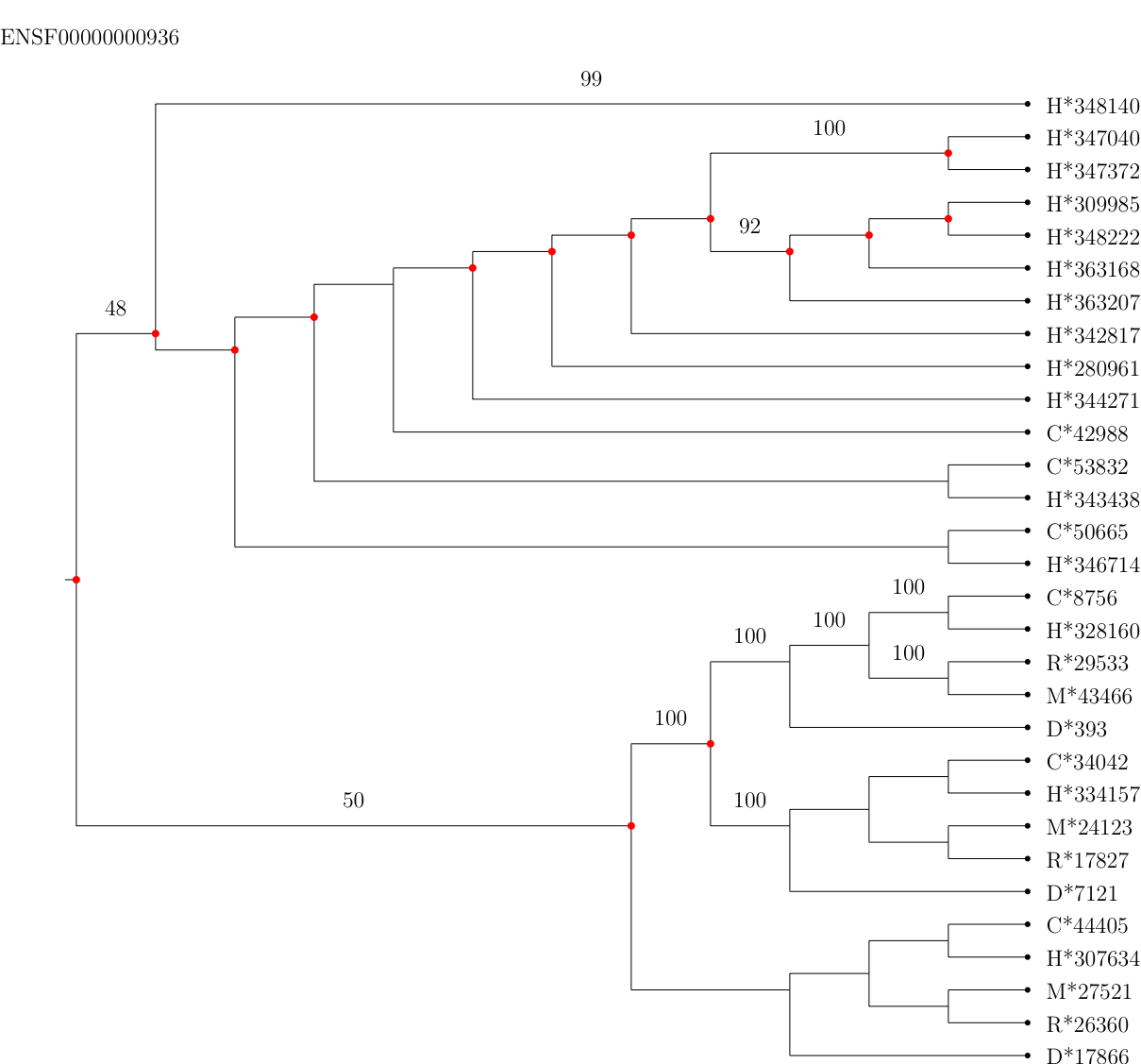

Supplement: Figure S4 — Gene tree for the golgin gene family (ENSF00000000597), showing gene duplication events as red boxes (H = human, C = chimp, M = mouse, R = rat, D = dog). (4.09 MB TIF) [file pone.0000085.s004.tif]
